# The risk of developing motor complications with Levodopa immediate versus dual release upon treatment initiation in Parkinson’s disease

**DOI:** 10.1016/j.prdoa.2023.100209

**Published:** 2023-07-04

**Authors:** Christian Ineichen, Heide Baumann-Vogel, Matthias Sitzler, Christian R. Baumann

**Affiliations:** Department of Neurology, University Hospital Zurich, University of Zurich, Frauenklinikstrasse 26, 8091 Zurich, Switzerland

**Keywords:** Parkinson’s disease, Motor complications, Levodopa immediate release, Levodopa sustained release

## Abstract

**Introduction:**

Motor complications (MCs) compromise therapy in many patients suffering from Parkinson’s Disease. By achieving more physiologic stimulation of dopamine-receptors, the continuous dopamine stimulation hypothesis suggests that longer-acting levodopa formulations may improve outcome. The aim of this study was to compare the duration until onset of MCs and motor disease progression in patients during their treatment initiation with either an immediate (IR) or a combined rapid- and sustained-release (i.e. dual-release; DR) levodopa formulation.

**Methods:**

Using a sample of 69 patients, we applied time-varying survival regression analyses and linear mixed effect models to analyze the data. The latter involved preprocessing of the data to temporally align the response and predictors, including analyzing the extent of visit irregularity and potential predictors of visit intensity.

**Results:**

This retrospective study suggests that levodopa-benserazide DR is not superior to levodopa-benserazide IR in affecting duration until MCs and disease progression. Conversely, using DR levodopa-benserazide, similar disease progression was achieved with lower and more constant doses.

**Conclusions:**

The effects of DR levodopa-benserazide might not be strong enough to delay onset of MCs. The development of more powerful levodopa formulations remains a pressing clinical need.

## Highlights

• DR levodopa-benserazide is not superior to IR levodopa-benserazide in affecting duration until motor complications.
• DR levodopa-benserazide is not superior to IR levodopa-benserazide in affecting motor disease progression.
• DR levodopa-benserazide was able to manage disease progression by using relatively low and constant doses.

## 1 Introduction

Levodopa’s significance in pharmacological treatment of Parkinson’s disease (PD), as a means to orally substitute striatal dopamine deficiency, has been reemphasized during the past years. Unfortunately, symptomatic efficacy and high tolerability are challenged by the perspective of long-term treatment complications, i.e. motor fluctuations and drug-induced dyskinesias. Accordingly, prospective double-blind studies have revealed that wearing-off hypokinesia and dyskinesia (i.e. cycling of patients between OFF-dystonia and peak-dose dyskinesia) may develop in up to 60% after 3 to 4 years and in almost all after 10 years of treatment with levodopa [1], [2]. On the other hand, the prevalence and severity of motor complications (MCs) appear to be similar in patients with early- or delayed levodopa treatment initiation [3].

From an aetiopathophysiological perspective, there are various factors likely contributing to the emergence of MCs, such as pathological changes to neural firing patterns, dopamine buffering capacities, or changes in pre- and postsynaptic dopaminergic transmission [4]. However, discontinuous drug delivery most probably plays a role in the pathophysiology of MCs [4], as pulsatile stimulation of dopamine receptors over time leads to aberrant network dynamics [5]. Apparently, repeated dopamine neuron activation through optogenetic stimulation can produce movements similar to levodopa induced dyskinesias and correspondingly, continuous dopaminergic stimulation (CDS) has shown to reduce the risk of MCs in both preclinical [4], [6] and clinical [7] settings.

The dual release (DR) levodopa formulation combines instant therapeutic benefits with longer-term effects of a delayed release profile [8]: To achieve a more controlled release profile compared to fast-release formulas [9], and to improve the wearing-off phenomenon and counterbalance the problem of unpredictable bioavailability of slow-acting release forms, Madopar® DR has been developed, consisting of three-layer tablets to obtain a first rapid increase in the plasma concentration of levodopa (comparable with standard Madopar® IR (immediate release)) and to maintain afterwards a plateau up to 4 h (comparable with Madopar® HBS (Hydrodynamically Balanced System)) [10], [11]. The peripheral decarboxylase inhibitor benserazide is provided with the initial release phase. In in vitro experiments and in in vivo pharmacokinetic studies these target characteristics were confirmed. Clinically, time to ON-state is improved by the DR formulation with duration in ON-state (and quality of ON phase) being similar compared to a slow-release formula [12]. Along the CDS hypothesis, dual- or sustained-release formulations might have a direct beneficial impact on the incidence or duration until onset of MCs. Unfortunately, much data on the emergence of MCs stems from cross-sectional studies or clinical trials on selected patient groups rather than population-based PD cohorts that have employed a direct comparison (summarized in [2]).

Therefore, with this retrospective single-center study in a population-based PD cohort, we compared the latency from treatment initiation until onset of MCs in patients initially treated with levodopa-benserazide (Madopar® IR, tab. 125 mg: Levodopa (100 mg), Benserazide (25 mg), ratio: 4:1, Roche Pharma) and those in whom treatment started with levodopa-benserazide DR (Madopar® DR, tab. 250 mg: Levodopa (200 mg), Benserazide (50 mg), Roche Pharma). Systematic data collection began in 2010 and comprises high primary data quality – a combination of clinical assessments of Parkinsonism and drug-related symptoms during visits through specialized neurologists, and patients’ self-assessments on MCs. In accordance with the CDS hypothesis, we hypothesized that onset of MCs will be delayed in the DR group.

## 2 Methods

We retrospectively analyzed data from PD patients ascertained from the movement disorders outpatient unit at the University Hospital Zurich (n = 69; IR group: 33, DR group: 36). Data were extracted from a large institutional database. The study was approved by the local ethics committee (No. 2021–01980), and patients provided informed consent. All subjects had a verified diagnosis of PD, information on scores of the MDS-UPDRS part III, its axial subscore, MDS-UPDRS part IV based neurological assessment of MCs combined with patient accounts (details below), levodopa equivalent daily dose (LEDD), fraction of dopamine agonists of LEDD, total number of daily drug intakes, PD subtype (akinetic-rigid, tremor-dominant, equivalent, other), gender and age. In addition, the number of patients with adjunct treatment using dopamine agonists (DA), dopamine reuptake inhibitors (monoamine oxidase B inhibitors (MAOBI) or catechol-O-methyltransferase inhibitors (COMTI)) was assessed (DA use, IR: n = 13 (39%), DR: n = 12 (33%); MAOBI use, IR: n = 11 (33%), DR: n = 11 (31%); COMTI use, IR: n = 2 (6%), DR: n = 3 (8%)) as well as their mean duration of intake (DAs, IR: 459 days, DR: 137 days [p < 0.05]; MAOBIs, IR: 151 days, DR: 221 days [p > 0.48]; COMTIs, IR: 6 days, DR: 24 days [p > 0.31]). At every visit MCs were evaluated. Based on clinical assessment (MDS-UPDRS Part IV items: time spent with dyskinesias, functional impact of dyskinesias, time spent in the OFF state, functional impact of fluctuations, complexity of motor fluctuations, painful OFF-state dystonia) and patients’ self-assessment, patients were categorized by motor fluctuations, and drug-related dyskinesias into MC status 0/1 (not occurring/occurring). To minimize the risk of missing any incident of onset of MCs, patient histories were checked individually for any indication of MCs (for further descriptions of MCs see e.g. [13]). Only longitudinally followed patients who fulfilled the criteria of stable treatment initiation were included (n = 69).

All early PD patients with either Madopar® IR or Madopar® DR at treatment initiation had an initial MDS-UPDRS III (motor symptoms) score of ≤ 24 and were treated with 562.5 mg or less LEDD. Patients who underwent DBS before or during the observation time were excluded from the analysis. For inclusion, Madopar® IR or DR treatment was maintained for at least 6 months after treatment initiation. One additional dopamine agonist or dopamine reuptake inhibitor was allowed. In the IR group, we allowed for a single Madopar® DR intake at night.

As primary objective, we compared the time from first visit with initiation of treatment (t = 0) until MCs (i.e. survival to onset of motor fluctuations or dyskinesias) among PD patients who received levodopa IR or DR treatment. To describe overall time-to-event data, we graphed the Kaplan-Meier curve. To increase model specificity (and account for violations to assumptions of proportional hazards), we included covariate interactions with time as predictors in the Cox model. Hence, a time-varying/dependent Cox proportional hazard model including potential confounders (time-varying/dependent confounders: MDS-UPDRS III, total LEDD, dose of dopamine agonists, total number of daily drug intakes; baseline or time-fixed confounders: sex, PD subtype, age) was used. Using the same methodology, time until onset of postural instability was investigated.

Finally, a longitudinal comparison of motor disease progression as assessed by the MDS-UPDRS part III and its axial subscore in habitual ON condition between treatment groups was performed. For longitudinal modelling, preprocessing of data was performed to temporally align the response and predictors according to [14], [15]: The extent of visit irregularity (mean proportions of patients with 0, 1 and > 1 visits per bin for variable bin widths) and potential predictors of visit intensity (Cox- model using the Anderson-Gill formulation) were investigated. Given these data, and after confirming that visit intensity was not associated with disease severity (p > 0.32) during the predefined follow-up time, the follow-up was fixed at 5 visits, and a 200 days bin size was used. Motor disease progression was analysed using a linear mixed effects repeated measures model with fixed effects of treatment-group (levodopa IR vs DR), and a random effect of patient ID. Drug-dose (total LEDD), sex, PD subtype and age were included as covariates. Statistical significance was set at p ≤ 0.05, statistical testing was conducted using Python and R (versions 3.7.6, 4.0.5, respectively).

## 3 Results

**Fig. 1:**
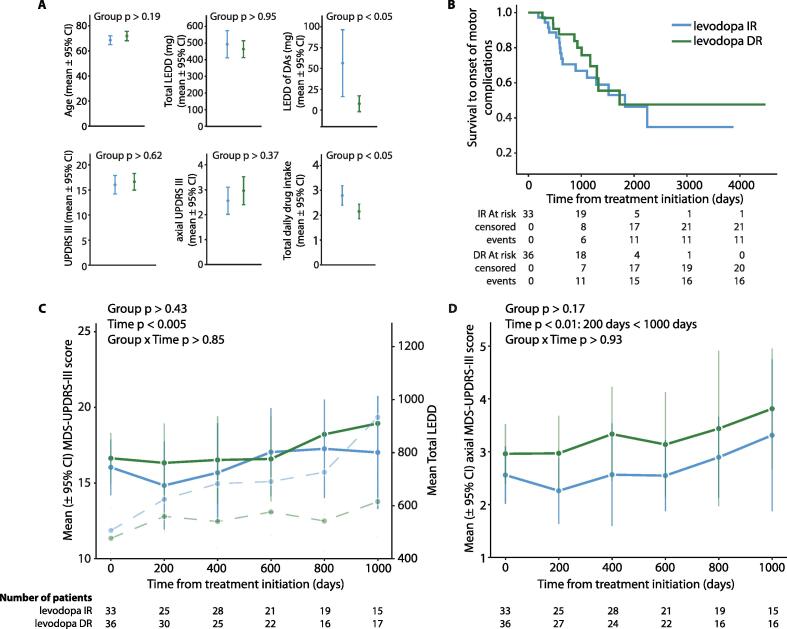
Survival to onset of motor complications and motor disease progression in patients during their treatment initiation with either IR or DR levodopa **A.** Baseline characteristics of patients of the DR- and IR-group for linear mixed models. For metric variables, either a *t* test or Mann-Whitney *U* test as the non-parametric alternative was used to compare differences between the two independent groups at treatment initiation: Upper: age, total levodopa equivalent daily dose (LEDD) and LEDD of dopamine agonists (DAs); lower: scores of the third (motor) part of the Unified Parkinson Disease Rating Scale of the International Parkinson and Movement Disorders Society (MDS-UPDRS III), its axial subscore and total number of daily drug intake. For categorical variables: % females: 42.4 vs 41.7 (p > 0.94); PD subtype (% akinetic-rigid/tremor-dominant/equivalent/other): 33/30/21/15 vs 31/28/33/8 (p > 0.63), IR vs DR, respectively; Chi-Square Test of Independence. **B.** Kaplan–Meier estimates of the probability that PD patients on dopaminergic therapy will be free from motor complications**. C & D.** Motor disease progression, as assessed by the MDS-UPDRS-III and the axial MDS-UPDRS-III, respectively, across time (1000 days) for the IR and the DR treatment group. In each graph, blue denotes to therapy using Madopar® IR whereas green represents treatment using Madopar® DR. In **C.**, the mean total LEDD along the 1000 days follow-up which was added as a time-dependent covariate in the statistical model, is additionally graphed (dotted lines; right y-axis). (For interpretation of the references to colour in this figure legend, the reader is referred to the web version of this article.)

IR patients were prescribed with significantly higher mean DA LEDD at treatment initiation (Fig. 1 A) and cumulatively over the observation period (IR: 665 mg, DR: 95 mg [p < 0.05]). Otherwise, there were no significant differences between groups regarding baseline patient characteristics. Kaplan-Meier survival estimates for treatment groups and time until MCs is presented in Fig. 1B. The time-varying Cox proportional hazards model for duration until MCs (Table 1) and duration until postural instability were used to compare treatment groups, adjusting for clinically important variables. Survival until MCs was not different in DR patients compared with the IR group (hazard ratio [HR], 1.06; 95% CI, 0.69–1.63; p > 0.80; median duration until MCs: IR-group: 1036 days, DR-group: 964.5 days), nor when restricting the follow-up to 1000 days. No covariates were significantly associated with time until onset of MCs. Regarding less levodopa-responsive axial symptoms, patients in the DR group failed to show significantly different survival until postural instability (hazard ratio [HR], 0.83; 95% CI, 0.47–1.45; p > 0.50; median duration until postural instability: IR-group: 945 days, DR-group: 799.5 days). Again, no covariates were associated with time until onset of postural instability. Fig. 1 C/D provide the results for the mixed effects repeated measures model displaying the motor disease progression for the MDS-UPDRS part III and its axial subscore along the nearly 3-year follow-up for the levodopa IR and DR groups. During treatment, motor disease progression was similar across groups (main effect of group; p > 0.43 and p > 0.17, respectively). Motor symptoms did increase significantly along the follow-up for the MDS-UPDRS III score and its axial derivative (main effect of time: p < 0.05; p < 0.01 – post-hoc: timepoint 200 days < timepoint 1000 days, respectively).

**Table 1:** Time-varying Cox proportional hazard regression analysis of predictor variables for development of motor complications.

Baseline variables	HR	95% CI lower	95% CI upper	p-value
DR-group	1.06	0.69	1.63	0.80
DA dosage	0.61	0.15	2.55	0.50
LEDD	1.00	1.00	1.00	0.63
Female sex	0.92	0.59	1.41	0.69
MDS-UPDRS III	1.01	0.99	1.04	0.29
Age	1.01	0.98	1.03	0.64
Daily_drug_intakes_total	0.97	0.83	1.13	0.69
PD (akinetic-rigid type)	0.92	0.58	1.48	0.74
PD (equivalent type)	0.83	0.51	1.36	0.46
PD (tremor-dominant type)	1.13	0.71	1.80	0.61

## 4 Discussion

This longer-term retrospective study in a population-based cohort compared patients stably treated with levodopa IR vs DR at treatment initiation and demonstrated that survival until MCs was similar in both groups. This is in line with a prior study showing that over a 5-year treatment period in *de novo* PD patients Madopar HBS was as effective as standard Madopar, but it showed no advantage in reducing motor fluctuations or dyskinesia [16]. In this study, however, Madopar IR was compared to Madopar HBS, which has a different pharmacokinetic profile. By comparing an immediate vs sustained release levodopa-carbidopa instead of a levodopa-benserazide combination (IR vs CR (Sinemet®), which is composed of levodopa and carbidopa in the ratio of 10:1) and by using a double-blind randomized study design, a further study provided evidence for therapeutic equivalence regarding the probability of developing motor fluctuations by 5 years [17] (for an overview on pharmacokinetics including immediate- and sustained release carbidopa‐levodopa and carbidopa‐levodopa‐entacapone see [18], [19]; for information on a novel carbidopa-levodopa extended release (Rytary®): [20]). To our knowledge, this is the first study investigating duration until onset of MCs using survival regression accounting for confounding factors and directly comparing immediate vs (a combined rapid- and) sustained-release levodopa.

Apparently, immediate- and sustained release levodopa tablets yield different pharmacokinetic profiles, and controlled-release formulations do not achieve CDS, but more sustained levodopa levels and smoothen plasma concentrations [4], [8]. This effect might not be strong enough to delay onset of MCs, as the literature has provided several other candidate factors impacting the risk of MCs in PD patients, for example aberrant network dynamics within the cortico-striato-thalamocortical and cerebello-thalamo-cortical circuits, or the neurodegenerative process [5], [21].

Similar to Kim et al [2], who analyzed the incidence of MCs in PD patients using a follow-up of up to 13 years, we found no significant relationship between levodopa dose (LEDD) and a higher risk to develop MCs. These results provide further support to the hypothesis that MCs are related to other factors rather than levodopa use, in accordance with a major study demonstrating a similar prevalence and severity of MCs in patients with early- or delayed levodopa treatment initiation [3].

Last not least, studies investigating different levodopa formulations on general measures of motor outcome are in line with the present results regarding motor disease progression insofar as they also demonstrated clinical equivalence (e.g. [22]). In our study, we fit both regression models using a time-dependent covariate thereby controlling for varying LEDDs. Even though there was no benefit in delaying onset of MCs when using levodopa DR, similar motor progression could be managed with significantly less LEDD (group main effect: p < 0.05), representing a minor but relevant clinical advantage over IR.

The study limitations pertain to the retrospective setting and the small sample size. Hence, these naturalistic retrospective observations need to be confirmed by prospective studies and await replication using a larger sample. The average follow-up duration was 3.3 years and studies with longer follow-ups may provide more conclusive data. On the other hand, using a relatively close follow-up, all data were ascertained in a structured and consecutive setting, with uniform and standardized outcome measures combined with patients’ self-assessment resulting in high-quality medical records suitable for assessing MCs for survival regression. Furthermore, defining the exact timing of onset of MCs is challenging, as motor fluctuations and dyskinesia often begin with subtle signs. The frequency of follow-up visits (6.5 ± 4.6 months (mean ± std)), however, should provide sufficient insights into the occurrence of MCs. To achieve feasibility, patients were included if stable IR vs DR therapy was maintained only over the first six months after treatment initiation. Still, we found that the mean interval on remaining on stable IR vs DR treatment until switch was 30.0 (IR: 26.5, DR: 33.6) months.

## CRediT authorship contribution statement

**Christian Ineichen:** Conceptualization, Data curation, Formal analysis, Methodology, Visualization, Writing – original draft, Writing – review & editing. **Heide Baumann-Vogel:** Data curation, Writing – review & editing. **Matthias Sitzler:** Data curation, Writing – review & editing. **Christian R. Baumann:** Conceptualization, Funding acquisition, Methodology, Writing – original draft, Writing – review & editing.

## Declaration of Competing Interest

The authors declare that they have no known competing financial interests or personal relationships that could have appeared to influence the work reported in this paper.
